# Targeting proteins for proteasomal degradation—a new function of *Arabidopsis* ABI1 protein phosphatase 2C

**DOI:** 10.3389/fpls.2015.00310

**Published:** 2015-05-05

**Authors:** Agnieszka Ludwików

**Affiliations:** Department of Biotechnology, Institute of Molecular Biology and Biotechnology, Faculty of Biology, Adam Mickiewicz University, Poznan, Poland

**Keywords:** ABI1, PP2C group A, ABA signaling, proteasomal degradation, stress signaling, phosphorylation, dephosphorylation

## Abstract

The ubiquitin/26S proteasome system (UPS) has been implicated in the regulation of many physiological processes including hormone signaling. The plant hormone abscisic acid (ABA) employs the UPS to control its own synthesis and signaling and to regulate stress response and tolerance. Among the known effectors of ABA signaling, the ABI1 (abscisic acid-insensitive 1) protein phosphatase, which belongs to group A of the type 2C protein phosphatases, is recognized as a key component of the pathway. Molecular and genetic evidence implicates this protein phosphatase in numerous plant responses. This mini-review discusses recent progress in understanding the role of ABI1 in ABA signaling, with particular emphasis on recent data that link ABI1 to protein degradation via the UPS.

Reversible protein phosphorylation is a key protein modification involved in the regulation of numerous physiological processes. Phosphorylation and dephosphorylation are catalyzed by protein kinases and protein phosphatases, respectively. In *Arabidopsis* there are over 1000 genes that encode protein kinases and protein phosphatases (Fuchs et al., 2013). These enzymes have critical functions in plant growth, development and stress responses, and ongoing research in *Arabidopsis* highlights the importance of protein phosphatases type 2C (PP2Cs) from group A as regulatory components of the ABA signaling pathway (Miyazono et al., 2009; Nishimura et al., 2009). Genetic screening experiments indicate nine group A PP2Cs (ABI1, ABI2, HAB1, HAB2, HAI1, HAI2, HAI3, PP2CA/AHG3, and AHG1) as negative regulators of ABA signal (Gosti et al., 1999; Merlot et al., 2001; Saez et al., 2004, 2006; Kuhn et al., 2006; Nishimura et al., 2007; Rubio et al., 2009; Fuchs et al., 2013). Clade A PP2Cs by interaction with multiple proteins enable a wide range of ABA responses in plants. Members of clade A PP2Cs interact with RCAR/PYR/PYLs (Nishimura et al., 2010; Hao et al., 2011; Antoni et al., 2012), SnRK1 (SnRK1.1-2; Rodrigues et al., 2013), SnRK2 (SnRK2.2-3, SnRK2.6; Umezawa et al., 2009), CBL-interacting protein kinases (CIPK8, CIPK14-15, CIPK20; Guo et al., 2002; Ohta et al., 2003; CIPK26, Lyzenga et al., 2013), the b-ZIP transcriptional factor (ATHB6; Himmelbach et al., 2002), and glutathione peroxidase (GPX3; Miao et al., 2006) to regulate ABA signaling or response. Apart from ABA signaling, group A of *Arabidopsis* PP2Cs have been involved in other pathways to regulate plant growth, development, ion transport and stress acclimation (Chérel et al., 2002; Himmelbach et al., 2002; Yang et al., 2006; Yoshida et al., 2006; Saez et al., 2008; Geiger et al., 2010; Ludwików et al., 2009, 2013, 2014).

Because ABI1 and ABI2 are best known for their key roles in ABA signaling, understanding how these phosphatase activities are regulated in response to ABA is one of the most important goals in plant research. In early studies, H_2_O_2_ was proposed to be a reversible inhibitor of ABI1 because of its inhibitory effects on ABI1-dependent phosphatase activity *in vitro* (Meinhard and Grill, 2001). However, more recent study does not support these findings, showing that *in planta* ABI1 maintains high phosphatase activity in response to oxidative stress conditions (Ludwików et al., 2014). Moreover, ABI1 is regulated by additional factors that increase or maintain its activity during stress conditions, such as the Rho-like small GTPases, which also protect ABI1 from ABA–PYL/PYR inhibition. Similarly, glutathione peroxidase (GPX3) and FERONIA receptor kinase play an important role in regulating the activity of ABI2 (Miao et al., 2006; Li et al., 2012; Yu et al., 2012; Ludwików et al., 2014).

In addition to the above regulators, the last years have seen notable progress in ABA receptor research. For example, the *Arabidopsis* PYR/PYL/RCAR family of START proteins have been identified as ABA receptors. PYLs comprise 14 members that fall into two distinct classes, dimeric and monomeric (Melcher et al., 2009; Miyazono et al., 2009; Nishimura et al., 2009; Santiago et al., 2009; Yin et al., 2009; Dupeux et al., 2011), with different affinities for ABA (Miyazono et al., 2009; Santiago et al., 2009; Yin et al., 2009; Szostkiewicz et al., 2010). The current model of the core ABA pathway assumes that ABA receptors in complex with ABA recognize and bind to PP2Cs, releasing SnRK2s from PP2C-dependent regulation (Melcher et al., 2009; Miyazono et al., 2009; Dupeux et al., 2011). Importantly, the above studies clearly demonstrated that PYR/PYL/RCAR receptors show preferences in substrate specificity and selectively inhibit specific PP2Cs in the presence of ABA, although one particular PP2C (AHG1) has been identified as insensitive to ABA-PYL inhibition (Antoni et al., 2012). On the other hand, PYL proteins differ in their ability to inhibit the phosphatase activity of group A PP2Cs even in the absence of the ABA ligand. PYR1 and PYLs 1–3 show only weak inhibitory effects on HAB1, while PYL4 shows clear inhibition of HAB2. PYL5–9 and PYL10 constitutively inhibit particular PP2Cs to a certain degree (Hao et al., 2011). Importantly, low-affinity complexes with PP2Cs generated in the absence of ABA are insufficient to activate ABA signaling (Antoni et al., 2012; Dupeux et al., 2011).

As well as the group A PP2Cs, an additional, underrated but essential component of ABA signaling has emerged in the last decade—the ubiquitin-proteasome system (UPS). The UPS requires the action of three types of enzymes: ubiquitin-activating enzymes (E1), a ubiquitin-conjugating enzyme (E2), and ubiquitin (Ub) ligases (E3). E3 Ub ligases determine the substrate specificity of the ubiquitination reaction and are classified into four groups: HECT, RING, U-box, and cullin-RING ligases (CRLs). Ub ligases function as either monomeric enzymes or multisubunit complexes. The largest and most diverse class of E3s are CRLs comprising the SCF (S-phase kinase-associated protein1-cullin1-F-box), the BTB (bric-a-brac-tramtrak-broad complex), the DDB (DNA damage-binding), and the APC (the anaphase-promoting complex/cyclosome) E3 Ub ligases (Vierstra, 2009; Chen et al., 2013; Stone, 2014).

Abscisic acid employs E3 Ub ligases in the management of its own synthesis and signaling to improve plant growth and development, as well as stress response and tolerance (Ko et al., 2006; Zhang et al., 2008; Raab et al., 2009; Salt et al., 2011; Chen et al., 2013; Lyzenga et al., 2013; Stone, 2014). Several ABA-responsive transcription factors, both positive and negative regulators of ABA signaling, including ABI3, ABI4, ABI5, ATHB6, ABF1, and ABF3, were found to be regulated by the UPS. The ABI3 transcription factor is polyubiquitinated by ABI3-interacting protein (AIP2), a RING-type E3 Ub ligase. ABA up-regulates AIP2 protein abundance, which in turn decreases ABI3 level. In addition, the *aip2-1* mutant is hypersensitive to ABA in root growth and seed germination assays. Thus AIP2 ligase is also regarded as a negative regulator of ABA signaling (Zhang et al., 2005). The opposite regulation is observed for the ABI5 transcription factor. ABA increases ABI5 abundance by supervision of KEG (KEEP ON GOING), a RING3-type E3 ligase that targets ABI5 for ubiquitination and subsequent degradation (Liu and Stone, 2010). In this case, ABA controls ABI5 levels by causing KEG to ubiquitinate itself and thereby promote its own destruction (Liu and Stone, 2010). Another regulator of ABI5 stability has been identified recently: ABA-hypersensitive DCAF1 (ABD1), which is a substrate receptor protein, modulates ABI5 turnover in the nucleus (Seo et al., 2014).

Similar mechanisms can be observed in the turnover of the ABF1, ABF3, and ATHB6 transcription factors (Himmelbach et al., 2002; Lechner et al., 2011). ABF1 and ABF3 are ABI5-related transcription factors and positive effectors of multiple ABA responses. Importantly, ABI5, ABF1, and ABF3 interact with ABI3, a transcription factor involved in seed maturation and dormancy, and ABF3 and ABI5 exhibit redundancy (Finkelstein et al., 2005; Chen et al., 2013). ABA probably affects ABF1 and ABF3 accumulation by preventing their degradation. Interestingly, proteolysis of ABF1 and ABF3 is affected by KEG—the same E3 Ub ligase that is involved in ubiquitination of ABI5. The mechanism of ABF1 and ABF3 function is complicated. The conserved C-terminal region (RRTLTGPW motif) required for interaction with 14-3-3 protein is also necessary for ABF1 and ABF3 stabilization. Although degradation of ABF1 and ABF3 is delayed in a *keg* mutant, KEG ubiquitinates both full-length ABF1/3 proteins and their C-terminal deletion forms. Authors postulate that ABF1 and ABF3 are stabilized by phosphorylation, probably driven by SnRK2 kinases. In addition, interaction with 14-3-3 proteins increases ABF1 and ABF3 stability (Chen et al., 2013).

As already mentioned, ABA prevents the turnover of ATHB6, a homeobox-leucine zipper transcription factor, which is another negative regulator of the ABA response and a target of ABI1 PP2C (Himmelbach et al., 2002; Lechner et al., 2011). ATHB6 directly interacts with, and is a target for degradation by, MATH-BTB proteins. The interaction between MATH-BTB proteins and ATHB6 occurs within the leucine zipper domain of ATHB6, suggesting that this interaction may interfere with the dimerization of ATHB6 with other HD-Zip proteins (Lechner et al., 2011). Furthermore, MYB30 transcription factor, a negative regulator of ABA signaling, is targeted for degradation by MIEL1, the RING-type MYB30-Interacting E3 Ligase 1 (Marino et al., 2013). MIEL1 attenuates cell death and pathogen resistance by promoting the destruction of MYB30.

The UPS is responsible for the rapid breakdown of ABA-regulated protein kinases (Fujii et al., 2009; Lyzenga et al., 2013; Rodrigues et al., 2013). Thus, KEG triggers the degradation of CIPK26, a sucrose non-fermenting-1 (SNF1)-related protein kinase 3 (SnRK3; Lyzenga et al., 2013). Although it has been demonstrated that CIPK26 is a component of ABA signaling downstream of ABI1, it is not clear whether ABI1 affects its kinase activity. Other ABA-regulated SnRKs have also emerged as targets for polyubiquitination (Lee et al., 2008; Fragoso et al., 2009). Importantly, regulation of the kinase activity of SnRK1 and SnRK2 by ABI1 and ABI1-like phosphatases has been clearly demonstrated (Yoshida et al., 2006; Rodrigues et al., 2013). The chloroplast localized SnRK1.2 is specifically degraded in response to phosphate starvation (Fragoso et al., 2009). SnRK1.1 and SnRK1.2 interact with PRL1 (Pleiotropic Regulatory Locus 1), a subunit of a CUL4-based E3 ligase (Bhalerao et al., 1999). In addition, both SnRK1 isoforms interact with SKP1 (S-phase Kinase-associated Protein1), a component of the CUL1-based E3 complex. Nevertheless, only SnRK1.1 has been shown to be degraded by the proteasome, with its breakdown orchestrated by a CUL4-based E3 ligase that uses PRL1 as a substrate receptor (Lee et al., 2008). Other members of the SnRK family, SnRK2.4 and SnRK2.6 (Kulik et al., 2011; Stone, 2014), as well other kinases involved in ABA signaling, including CDPK2, CDPK6, MPK3, and MPK4 (Ichimura et al., 2000; Lu et al., 2002; Mori et al., 2006; Danquah et al., 2014), are also known to be targets for ubiquitination (Kim et al., 2013).

Last but not least, the PYR/PYL/RCAR ABA receptors are directed to the UPS by the proteins De-etiolated1 (DET1) and DDB1-associated1 (DDA1), both of which assemble with CUL4-based E3 Ub ligase (CRL4; Chen et al., 2013; Irigoyen et al., 2014; Figure 1A). DET1 is known as a central regulator of photomorphogenesis and thermomorphogenesis (Delker et al., 2014; Dong et al., 2014; Li et al., 2015; Ly et al., 2015). Very recently, DET1 was identified as a central repressor of light-induced seed germination that controls the stability of phytochrome interacting factor 1 (PIF1) and long hypocotyl in far-red 1 (HFR1) proteins (Shi et al., 2015). Although it is unclear how DET1 controls the stability of ABA receptors, DDA1 binds PYL4 and PYL8-9, and mediates recognition by CRL4. ABA inhibits the degradation of ABA receptor PYL8 by limiting its polyubiquitination. Nevertheless, the exact mechanism of this regulation is unknown (Irigoyen et al., 2014).

**FIGURE 1:**
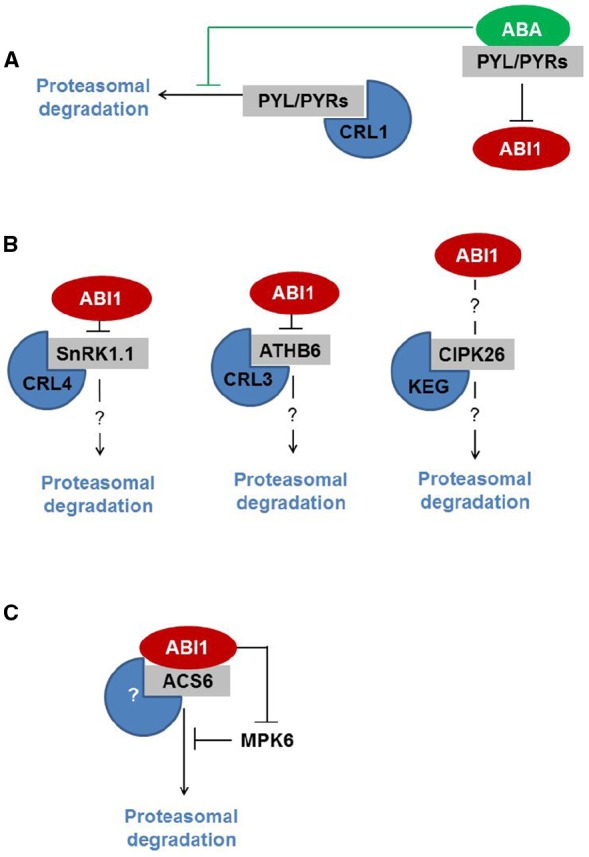
**ABI1 interacts with proteins that are subjected to UPS-mediated proteolysis. (A)** ABI1 interacts with ABA-bound PYL/PYRs, which inhibits its phosphatase activity. ABI1 downstream targets are therefore not subjected to degradation. **(B)** ABI1 interacts with SnRK1.1 and ATHB6, thereby modifying downstream signaling. SnRK1.1 and ATHB6 are degraded by CRL4- and CRL3- E3 Ub ligases, respectively. The role of ABI1 in the regulation of SnRK1.1 and ATHB6 is unknown. CIPK26, which is degraded by KEG, also interacts with ABI1. It is largely unclear how ABI1 regulates CIPK26-dependent signaling. **(C)** ABI1 interacts with ACS6. ABI1-mediated dephosphorylation targets both proteins for degradation by an unknown E3 Ub ligase. ABI1 also regulates the stability of ACS6 by affecting MPK6 kinase activity.

In the context of ABA regulators that are degraded by UPS, two common factors can be seen. Firstly, some UPS targets (like protein kinases and transcription factors) require phosphorylation for functional activation. In general, this is in line with the notion that phosphorylation and proteolysis cross-talks and are essential for ABA signal processing (Liu and Stone, 2010; Stone, 2014). Secondly, some ABA regulators (like ATHB6, SnRKs, and PYR/PYL/RCAR) are indeed the targets of ABI1 protein phosphatase (Himmelbach et al., 2002; Yoshida et al., 2006; Lyzenga et al., 2013; Rodrigues et al., 2013; Irigoyen et al., 2014; Figure 1B). So we might ask: does ABI1 contribute to the regulatory mechanism?

Following the recent report on the role of ABI1 in protein turnover, the answer to this question is positive (Figure 1C). Ludwików et al. (2014) show that under ozone stress conditions, type I ACC synthase (ACS) turnover is controlled by ABI1 at two levels: (i) ABI1 dephosphorylates ACS6 at the C-terminally located MPK6 target-site, thereby promoting ACS6 degradation; (ii) ABI1 inhibits MPK6 activity directly in this way modifying the phosphorylation rate of the ACS6 protein. Based on this report we might hypothesize that ABI1, and possibly more group A PP2Cs, target and control the turnover of other downstream regulators of the ABA signaling pathway.

In conclusion, UPS-mediated proteolysis seems to be a prominent mechanism for removing certain dephosphorylated ABA-signaling elements from the cell. In the context of recent studies on ABI1 (Figure 1), the negative regulation of ABA signaling by ABI1 takes on new meaning: ABI1 resets various signaling pathways to pre-stimulatory status by targeting downstream regulators for degradation by UPS. The future challenge in this research area undoubtedly lies in the identification of ABA-pathway regulators that are controlled by the UPS. Finally, another significant task will be to understand how ABI1 recognizes the protein targets that must be degraded. We might assume that ABI1 binds a particular protein motif, whose dephosphorylation leads to destruction of the target protein; however, currently no such motif is known. Nevertheless, the interplay between ubiquitination and phosphorylation has emerged as a key mechanism regulating protein stability (Yoo et al., 2008; Spoel et al., 2009; He and Kermode, 2010; Wang et al., 2012; Nguyen et al., 2013; Zhai et al., 2013). Whether ABI1 recognizes signals that are encoded in patterns of posttranslational modifications—we are about to learn.

## Conflict of Interest Statement

The author declares that the research was conducted in the absence of any commercial or financial relationships that could be construed as a potential conflict of interest.
